# Association of interleukin-1 beta (-511C/T) polymorphisms with osteoporosis in postmenopausal women

**DOI:** 10.4103/0256-4947.71062

**Published:** 2010

**Authors:** Tai-Hung Chao, Hsing-Ning Yu, Chi-Chuan Huang, Wen-Shen Liu, Ya-Wen Tsai, Wen-Tung Wu

**Affiliations:** a From the Department of Orthopedics, Zuoying Armed Forces General Hospital, Kaohsiung, Taiwan; b From the Asia-Pacific Biotech Developing, Inc., Kaohsiung, Taiwan; c From the Department of Biotechnology, Yung-Ta Institute of Technology & Commerce, Pingtung, Taiwan

## Abstract

**BACKGROUND AND OBJECTIVES:**

Osteoporosis is a common disease of the elderly, in which genetic and clinical factors contribute to the disease phenotype. Since the production of interleukin-1 (IL-1) has been implicated in the bone mass and skeletal disorders, we investigated whether IL-1 system gene polymorphisms are associated with the pathogenesis of osteoporosis in postmenopausal Taiwanese women.

**METHODS:**

Osteoporosis is diagnosed by dual-energy x-ray absorptiometry, which measures bone mineral density (BMD) at multiple skeletal sites. We studied the IL-1α (-889C/T), IL-1β (-511C/T) and the 86 base pair variable number tandem repeat (VNTR) in intron 2 of the IL-1 receptor antagonist (IL-1ra) gene in 117 postmenopausal women with osteoporosis and 135 control subjects without a history of symptomatic osteoporosis. These gene polymorphisms were analyzed by polymerase chain reaction and restriction fragment length polymerase. Blood sugar and other risk factors were also determined.

**RESULTS:**

The frequencies of IL-1β (-511C/T) genotypes (*P*=.022, odds ratio=1.972) and alleles (*P*=.02, odds ratio=2.909) showed a statistically significant difference between the two groups. However, we did not find any statistically significant difference in IL-1α and IL-1ra polymorphisms (*P*>.05). We also observed a positive relationship between osteoporosis and cholesterol and a weak inverse relationship between blood sugar and osteoporosis in postmenopausal women.

**CONCLUSIONS:**

These experimental results suggest that the pathogenesis of osteoporosis is associated with IL-1β (-511C/T) polymorphism in postmenopausal women. This polymorphism is an independent risk factor for osteoporosis.

Osteoporosis is a common skeletal disorder among the elderly. Symptomatic osteoporosis is due to a reduction in bone mineral density (BMD), which is a risk factor for bone fracture. Low BMD values are strongly associated with osteoporotic fracture. Approximately 50% of all women suffer from osteoporosis; in these women, BMD values fall progressively with age. Several risk factors contribute to osteoporotic fracture, such as advanced age, low body mass index, previous fracture, muscle weakness and a family history of fracture.^1^

A previous study reported that interleukin-1 (IL-1) and IL-1 receptor antagonist (IL-1ra) gene polymorphisms were the most important genetic factors related to bone mass in postmenopausal women.^2^ The osteoblast, a relative of the macrophage, is a bone-resorptive cell that contributes to bone formation. The growth of the osteoclast and macrophages are mediated by the IL-1 system.^3^ If bone resorption exceeds bone formation, the risk of bone loss and osteoporotic fracture increases. The IL-1 system consists of three proteins: IL-1α and IL-1β are pro-inflammatory proteins, and IL-1ra is an antagonist protein. These proteins are encoded by the genes IL-1α, IL-1β and IL-1ra, respectively.^4^ IL-1α and IL-1β are known to be potent stimulators of bone resorption. IL-1ra is a competitive inhibitor of IL-1, as it bonds to the same receptor sites as does IL-1. Both proteins stimulate the proliferation and differentiation of osteoclast precursors into mature osteoclasts.^5^,^6^

Kim et al showed that there was an association between bone mass and IL-1 system polymorphisms in postmenopausal Korean women.^7^ The polymorphisms of IL-1 system, the IL-1α (-889 C/T), IL-1β (-511 C/T) and IL-1ra (86-bp VNTR), have been shown to be strong heritable components,^8^–^10^ but only a few studies have examined the possible relationships between the IL-1 system polymorphisms and osteoporosis, and the results are controversial.^11^–^14^ The relationship between IL-1 system and osteoporosis needs to be elucidated for different populations.

In the study, we hypothesized that IL-1 system polymorphism might be associated with the pathogenesis of osteoporosis. In particular, inheritance of genotype or allele variations in the cytokines genes may predispose to the risk of osteoporosis. To investigate this hypothesis, the polymorphisms of IL-1α (-889 C/T), IL-1β (-511 C/T) and IL-1ra (86 bp VNTR) were analyzed in patients with osteoporosis and in normal controls. Additionally, we examined the association of serum triglycerides and total cholesterol with osteoporosis in postmenopausal women.

## METHODS

### 

Postmenopausal women with or without osteoporosis who were not taking hormone therapy were recruited from the Zuoying Armed Forces General Hospital in Taiwan from March to October in 2008. All individuals agreed to participate in the study. Clinical laboratory examination included age, body mass index, plasma glucose, triglycerides and cholesterol. The study plan was accepted and supported by the ethics committee of Zuoying Armed Forces General Hospital. All specimens were collected and stored at –20°C until DNA extraction.

Osteoporosis was defined by a BMD value lower than 2.5 standard deviations below the young adult mean, according to World Health Organization’s criteria.^15^ BMD was measured by the dual-energy x-ray absorptiometry in grams per centimeter at the lumbar spine and the hip (a total hip or femoral neck).^16^ The study excluded all secondary causes of osteoporosis, such as hyperparathyroidism, tumors, or surgical menopausal status.

#### DNA extraction

Total genomic DNA was extracted with the DNeasyTM kit (Qiagen, USA) according to the manufacturer’s instructions. Briefly, the blood was digested with 0.5 mg/mL proteinase K in 400 μL cell-lysis solution for 24 hours at 55°C until the blood was completely lysed. After adding 200 μL absolute ethanol to the lysed sample, the mixture was transferred into the DNeasy mini column and centrifuged for 1 minute at 8000 rpm. The DNeasy mini column was washed with 500 μL washing buffer and centrifuged for 1 minute at 8000 rpm. Finally, the DNA was eluted in a clean 1.5-mL microcentrifuge tube. The amount of DNA was measured spectrophotometrically using a spectrophotometer (GeneQuant) and stored at –20°C until polymerase chain reaction (PCR) amplification.

#### Determination of IL-1 system gene polymorphisms

The C/T substitution at position -889 in the promoter region was assessed by PCR amplification, and the products were digested. The sequences of PCR primers were 5’ GCA TGC CAT CAC ACC TAG TT 3’ (sense) and 5’ TTA CAT ATG AGC CTT CCA TG 3’ (antisense) with an expected PCR product size of 194 bp (from –1062 to –869). Amplification was performed by using a Perkin-Elmer 9700 thermal cycler (Applied Biosystems, Foster City, CA) and polypropylene PCR plates no. 170651 (Biozym, Landgraaf, The Netherlands). The amplification conditions consisted of 94°C for 3 minutes, followed by 45 cycles of 94°C for 1 minute, 56°C for 1 minute and 72°C for 40 seconds. The reaction was terminated by a final elongation at 72°C for 7 minutes. The products were digested with 5 U/μL of NcoI at 37°C for 4 hours and formed 178- and 16-bp DNA products for allele C (allele 1 or wild-type allele) and an intact fragment of 194-bp DNA products for allele T (allele 2 or variant allele). The digested products were separated on a 4% agarose gel and then stained by ethidium bromide (0.5 μg/mL), and genotypes were determined by analyzing different bands. To ensure accuracy, each test was performed three times for each sample. The fragments were to assess the genotypes 1.1, 1.2 and 2.2.

The C/T substitution was located at position -511 in the promoter region of the IL-1β gene. This region was amplified by PCR, using the primers 5’ TGG CAT TGA TCT GGT TCA TC 3’ (sense) and 5’ GTT TAG GAA TCT TCC CAC TT 3’ (antisense) (Invitrogen Life Technologies, Breda, The Netherlands) as described previously.^8^ Amplification was performed using a Perkin-Elmer 9700 thermal cycler (Applied Biosystems, Foster City, CA) and polypropylene PCR plates no. 170651 (Biozym, Landgraaf, The Netherlands). The following parameters were used: 94°C for 5 minutes, followed by 45 cycles of 94°C for 1 minute, 55°C for 1 minute and 72°C for 1 minute and a final incubation at 72°C for 7 minutes followed by a cooling to 4°C. The PCR products were analyzed by electrophoresis on a 2% agarose gel stained with 0.1% ethidium bromide. The 305-bp fragments were digested overnight at 37°C with 0.13 U/μL AvaI (New England Biolabs, UK), resulting in fragments that either remained intact (allele 2 or variant allele) or were cut into two fragments of 190 and 114 bp, respectively (allele 1 or wild-type allele). These fragments were analyzed by electrophoresis on 2% agarose gel containing 0.1% ethidium bromide to assess the genotypes 1.1, 1.2 and 2.2.

The polymorphic region, containing 86 base pair variable number of tandem repeats (VNTR) within the intron 2 of the IL-1ra gene, was amplified by PCR with the primers Gloria 1, 5’ CTC AGC AAC ACT CCT AT 3’ (sense); and Gloria 2, 5’ TCC TGG TCT GCA GGT AA 3’ (antisense) (Invitrogen Life Technologies).^10^ Amplification was performed by using a Perkin-Elmer 9600 thermal cycler (Applied Biosystems) and polypropylene thin-wall tubes no. 179501 (Biozym). The parameters were, an initial denaturation at 94°C for 5 minutes, followed by 45 cycles of denaturation at 94°C for 1 minute, annealing at 56°C for 1 minute and elongation at 72°C for 1 minute. The final elongation was at 72°C for 5 minutes followed by cooling to 4°C. The PCR products of 412 bp (A1=four repeats of the 86-bp region), 240 bp (A2=two repeats), 498 bp (A3=five repeats), 326 bp (A4=three repeats), 584 bp (A5=six repeats) and 756 bp (A6=eight repeats) were analyzed by electrophoresis on standard 2% agarose gel stained with 0.1% ethidium bromide.

#### Statistical analysis

Demographic and clinical data were compared between groups by analysis of variance (ANOVA). Genotype and allele frequencies were compared by the chi-square test for small sample size. The *P* values, odds ratios and 95% confidence intervals were calculated. A *P* value of less than .05 was considered significant for all analyses.

## RESULTS

We recruited 252 postmenopausal women (aged over 55 years), who were free of chronic diseases and medications known to affect bone formation, including 117 postmenopausal women with osteoporosis, aged between 56 and 74 years (mean age and standard deviation, 65.0 [7.69] years), and 135 postmenopausal women who did not have osteoporosis, aged between 55 and 78 years (mean age and SD, 65.8 [9.49] years) (**Table 1**). Differences in cholesterol and blood glucose levels were significantly different, but there were no significant differences in body mass index and triglycerides.

**Table 1:** Demographic and clinical data for normal and osteoporotic postmenopausal women.

	Osteoporosis (n=117)	Normal (n=135)	*P*
Age (years)	65.0 (7.69)	65.8 (9.49)	NS
Body mass index (kg/m^2^)	24.8 (2.71)	26.3 (12.22)	NS
Triglyceride (mg/dL)	110.8 (62.82)	101.0 (78.87)	NS
Cholesterol (mg/dL)	190.2 (22.63)	148.1 (47.67)	.001
Blood glucose (mg/dL)	99.2 (16.01)	120.0 (48.81)	.043

Values are mean (standard deviation)

There was a significant association between the IL-1β (-511C/T) gene and osteoporosis (**Table 2**). Among 117 osteoporosis patients, 30 (25.6%) had the CC genotype compared to 18 (13.3%) of the 135 women without osteoporosis (*P*<.05). Statistically significant differences were found in allele frequency for IL-1β when osteoporosis patients were compared with non-osteoporotic women (*P*<.05). These results indicate that the individuals carrying the genotypic variation of IL-1β (-511C/T) have an increased risk of having osteoporosis.

**Table 2:** Genotype and allele frequencies of IL-1 system genotypes in non-osteoporotic and osteoporotic postmenopausal women.

Gene	Genotype	Allele	Osteoporosis (n=117)	Normal (n=135)	OR(95% CI) P	*P*
IL-1α -889					0.952 (–0.211 - 0.337)	NS
	CC		93 (79.6%)	114 (84.4%)		
	CT		12 (10.2%)	9 (6.7%)		
	TT		12 (10.2%)	12 (8.9%)			
IL-1β -511					1.972 (–0.692 - –0.056)	.022
	CC		30 (25.6%)	18 (13.3%)		
	CT		54 (46.2%)	45 (33.3%)		
	TT		33 (28.2%)	72 (53.4%)		
IL-1ra					0.988 (–0.167 - 0.170)	NS
	A2		21 (17.9%)	24 (17.8%)		
	A1		93 (82.1%)	111 (82.2%)		
IL-1α-889					0.714 (–0.369 - 0.201)	NS
		C	198 (84.6%)	237 (87.7%)		
		T	36 (15.3%)	33 (12.2%)		
IL-1β-511					2.909 (0.042 - 0.472)	.020
		C	114 (48.7%)	81 (30.0%)		
		T	120 (51.2%)	189(70.0%)		

OR: odds ratio, CI: confidence interval

## DISCUSSION

Bone mineralization is associated with differentiation of the osteoblast, which secretes osteopontin, one of the bone matrix proteins involved in bone formation. Rajamannan et al^17^ found that expression of osteopontin was stimulated by a high cholesterol diet, since cellular proliferation and bone matrix production were found to be mediated by hypercholesterolemia in in vivo experiments. Kha et al^18^ suggested that oxysterols, the products of cholesterol oxidation, contribute to the regulation of stem cell differentiation toward osteoblasts. A study by Brownbill et al^19^ showed that higher levels of serum lipids are positively associated with higher bone mineral density in postmenopausal women. Dennison et al^20^ demonstrated a relationship between BMD and lipids, but no relationship for BMD with total or LDL cholesterol. However, several studies have reported the opposite relationship, that lower BMD or osteoporosis is associated with higher total cholesterol levels in postmenopausal women.^21^,^22^ We also found a positive relationship between high cholesterol levels and osteoporosis and a weak inverse relationship between blood glucose and osteoporosis, but no relationship between osteoporosis and serum triglycerides and BMI in this study. Similarly, Samelson et al^23^ found an inverse relationship between elevated total cholesterol and lower BMD. These contradictory reports indicate that the mechanism of this association between lipid profile and BMD needs to be elucidated. They also lead to the conclusion that cholesterol is not a long-term clinical factor contributing to osteoporosis.

Osteoporosis is a common disease of the elderly, suggesting a possible link among various factors, including dietary saturated fat, physical activity, medical treatment and metabolic disorders. Osteoporosis is a polygenic disorder and is affected by the expression of several genes in the regulation of bone formation and osteoporotic fractures; for example, hormones and receptors, such as the vitamin D receptor (VDR) and estrogen receptor (ER); cytokines and receptors, such as IL-1α, IL-1β and IL-1ra, and transforming growth factor β1.^23^–^27^ Among genetic factors in the pathogenesis of osteoporosis, the IL-1 system was found to be the most important cytokine in modulating the growth of bone-resorptive cells in postmenopausal women.^27^–^30^ The IL-1 system is composed of IL-1α, IL-1β and IL-1ra. Both IL-1α and IL-β bond to the IL-1 receptor on the surface of blood cells, and they initiate a cascade of signal transduction to stimulate a potent pro-inflammatory response that initiates bone resorption. IL-1ra also bonds to the same IL-1 receptor, but is a competitive inhibitor of IL-1. However, these results are not consistent in different populations. For example, Langdahl et al^12^ reported that osteoporotic fractures were associated with IL-1ra, but not with polymorphism of IL-1β gene in whites. Kim et al^31^ reported that the association between IL-1ra VNTR polymorphism and BMD was identified in postmenopausal Korean women. Nevertheless, Bajnok et al^11^ observed a lack of association between the IL-1ra gene polymorphism and BMD in postmenopausal Hungarian women. Thus, the association between BMD and IL-1 cytokines is still unclear.^32^

In our study, the allelic and genotypic frequency results showed that IL-1β genotype was more frequent in postmenopausal Taiwanese women with osteoporosis. However, no significant differences were found in the distribution of IL-1α and IL-1ra genotype. This result is consistent with that of previous studies. For instance, Nemetz et al^13^ reported that allelic variation at the IL-1β gene was associated with reduction of bone mass in patients with inflammatory bowel disease. Chen et al^14^ reported similar findings, that IL-1β and IL-1ra gene polymorphisms were associated with BMD and osteoporosis in postmenopausal women.

In summary, the IL-1β polymorphism was found to be a genetic factor that might influence the maintenance of bone mass in postmenopausal Taiwanese woman. The relationship between IL-1α/ IL-1ra and bone mass is still unclear. Therefore, it is essential to carry out further studies in larger populations and other ethnic groups. Our experimental results suggest that a primary genetic analysis be part of a proper consultation so that precautions can be given to patients on familial genetics.
